# Congenital Clasped Thumb That Is Forgetten a Syndrome in Clinical Practice: A Case Report

**DOI:** 10.1097/MD.0000000000001630

**Published:** 2015-09-25

**Authors:** Sancar Serbest, Haci Bayram Tosun, Ugur Tiftikci, Seyit Ali Gumustas, Abuzer Uludag

**Affiliations:** From the Faculty of Medicine, Department of Orthopedics and Traumatology, Kırıkkale University, Kırıkkale, Turkey (SS, UT) and Faculty of Medicine, Department of Orthopedics and Traumatology, Adiyaman University, Adiyaman, Turkey (HBT, SAG, AU).

## Abstract

Congenital clasped thumb is a progressive flexion and adduction deformity presenting with heterogeneous congenital anomalies. Although the disease is rare, diagnosis is usually delayed due to natural location of thumb within the palm in first 3 months of life.

A 4-year-old girl with congenital clasped thumb deformity due to absence of extensor pollicis brevis tendon whose treatment consisted of extensor indicis proprius (EIP) transfer and z-plasty reconstruction to first web space.

The patient was so happy with both cosmetic appearance and functional status. There was not any limitation at interphalangeal or metacarpophalangeal (MCP) joints of the thumb and the result was. Stability of MCP joint was full and power for grasping any object was much better than the original status.

In cases of isolated clasped thumb deformity associated with absence of tendon whose treatment attempts with splinting and physical treatment have failed, EIP tendon transfer and reconstruction of contracture in first web space with z-plasty is an easy and successful method to obtain functional improvement.

## INTRODUCTION

Congenital clasped thumb is a progressive flexion and adduction deformity presenting with heterogenous congenital abnormalities.^1^ In this rare syndrome, main problem is the deficient extensor tendon mechanism of thumb due to functional and structural causes.^2–4^ It is confused with trigger finger deformity due to fixated flexion deformity of the thumb In congenital trigger finger entrapment is present at A1 pulley level due to fusiform enlargement of flexor pollicis longus tendon,^2,5,6^ although in congenital clasp finger deformity there are thumb extension deformity due to extensor tendon insufficiency and muscle and skin contractions at flexor and thenar regions.^3,4,7^

In congenital clasped thumb success of treatment depends on lesion type. Although successful results have been obtained with splinting and conservative treatment several reconstruction methods are used due to secondarily developed contractures in neglected cases. Tendon transfers are useful in hypermobile thumb cases who have both extensor and opponents tendon deficiency.^8,9^

Aim of this study was to discuss a case with congenitally clasped thumb deformity who did not have extensor pollicis brevis (EPB) tendon and had contracture in first web space under the light of literature.

## CASE REPORT

**FIGURE 1:**
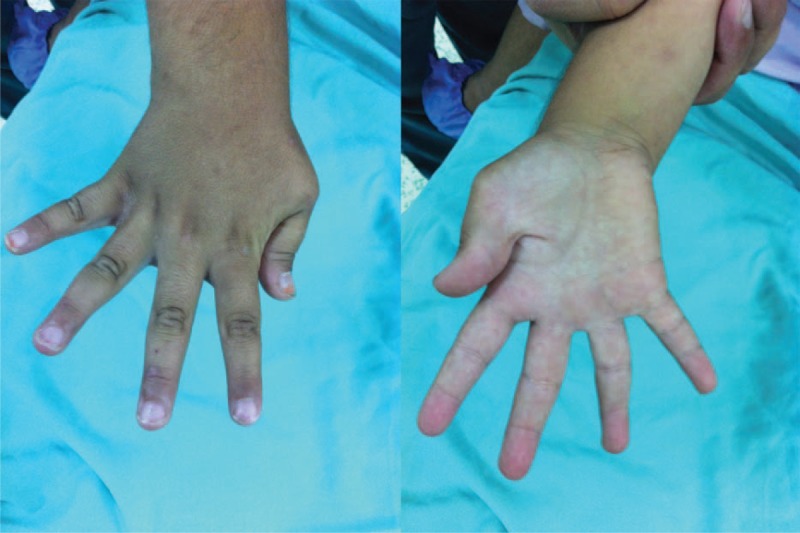
Adducted and flexed thumb before surgery.

**FIGURE 2:**
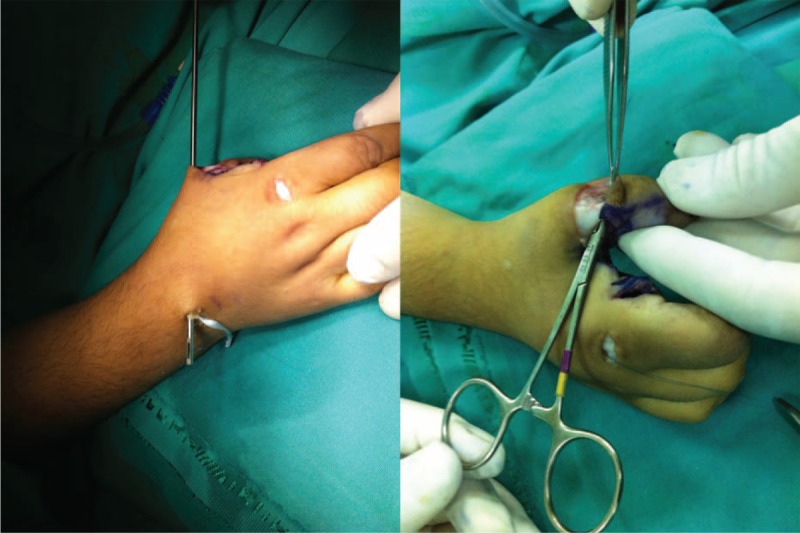
During surgery, construction of a tunnel in proximal phalanx, passage of extensor indicis tendon from this tunnel, and z-plasty reconstruction to first web interval.

**FIGURE 3:**
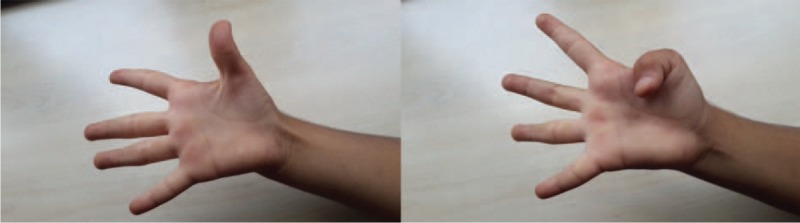
Appearance of hand and thumb 2 years after surgery.

A 4-year-old female presented to our clinic with limitation of movement at right-hand first finger. Physical examination revealed adduction and flexion deformity at right-hand first finger. When she was asked to make abduction and extension with right thumb range of motion for flexion and extension at interphalangeal (IP) joint was full but extension and abduction at metacarpophalangeal (MCP) joint was limited (Figure 1). There was not any sign of general instability at MCP joint like laxity of ulnar collateral ligament and passive finger extension was limited due to contraction at first web interval. Her other skeletal system examinations were normal. Family history revealed arthrogryposis and clasped thumb deformity in both hands in his 14-year-old brother. Due to absence of EPB tendon we performed extensor indicis proprius (EIP) tendon transfer and simple z-plasty reconstruction to first web space. We entered with a mini incision at second MCP joint from the dorsum of the hand. EIP tendon was found and tenotomized, pulled to wrist level, and then transferred to thumb with a subcutaneous tunnel. Then a bone tunnel was opened to proximal phalanx that fits to the original insertion place of EPB tendon and transferred EIP tendon was embedded to this tunnel and fixated (Figure 2). To protect the transferred tendon, fixation with a K wire for 6 weeks and a splint were applied. At 6th week after the surgery the K-wire was removed and a splint which holds thumb at extension and abduction position for 6 months after the surgery was applied. Daytime active exercises to achieve extension power of the thumb were prescribed. At her last control 2 years after the surgery thumb range of motion was full (Figure 3). The patient was so happy with both cosmetic appearance and functional status. There was not any limitation at IP or MCP joints of the thumb and the result was excellent according to Weckesser et al^8^ staging. Stability of MCP joint was full and power for grasping any object was much better than the original status. According to Gilbert classification abduction was 40° to 45°, rotation was 110° to 120°, and opposition of thumb with all other fingers was full.^10^ We informed both the patient and patient's parents about this study and obtained written informed consent.

## DISCUSSION

Congenital clasped finger is a syndrome characterized by loss of extension function of thumb due to absence of 1 or both of EPB and longus tendons.^2–4^ Several classification systems exist to categorize several spectrums of congenital clasped thumb. McCarroll^11^ and Tsuyuguchi's^12^ classification is among the most commonly used ones. Tsuyuguchi et al^12^ separated this syndrome into 3 groups. Type 1 is the flexible type in which thumb can be passively extended with no other abnormality. In type II thumb cannot be passively abducted or extended, and skin contracture, and there are comorbid collateral ligament or thenar muscle abnormalities. Type III occurs together with arthrogryposis.

Weckesser et al^8^ proposed a staging system which resembles Tsuyuguchi classification and classified into 4 groups according to the severity of deformity. In group 1, there is only limitation in extension of thumb; in group 2, flexion contractures are present in addition to deformity in group 1. Group 3 is hypoplastic thumb in which there are diffuse changes in all structures of thumb and flexor and thenar muscles are hypoplastic. Group 4 includes more complicated cases that do not fit into any of the groups. Our case was type II according to Tsuyuguchi classification and type III according to Weckesser et al classification.

This condition is usually confused with trigger finger due to fixated flexion deformity. Whether trigger thumb is congenital or not is controversial and generally there is no accompanying additional pathology.^13^ On the other hand, congenital clasped thumb deformity is generally bilateral, characterized by familial tendency and congenital lack of extensor mechanism, and is much rare than trigger finger.^6^ In congenital trigger, finger nodularity (Notta's node) is palpated at MCP joint level and it is difficult to passively extend IP joint. Although real triggering cannot be observed, forced extension of thumb may produce pain and a click sound. On the other hand, flexion deformity can be corrected passively but not actively in congenital clasped thumb deformity.^2,5,6^

Ruland and Slake^7^ reported that flexion deformity in clasped thumb syndrome may be confused with trigger finger and this may lead to unnecessary releasing surgeries. This approach aggravates symptoms instead of relieving them.

Diagnosis is difficult in first 3 to 4 months because a newborn naturally holds his thumb inside his palm in first 3 to 4 months. But when his cooperation with surroundings increase flexion posture starts to be prominent.^7,13^ At first evaluation, thumb is seen at flexion position, it cannot be extended, and after forced extension it returns to original flexion position. This deformity is generally together with other generalized musculoskeletal malformations.^13^

Treatment of congenitally clasped thumb depends on disease stage, age at presentation, and comorbid pathologies.^10,12^ Tsuyuguchi et al^12^ reported that good results were achieved with type I and type II cases with conservative methods. In type II and type III, cases in whom conservative treatment is ineffective surgical treatment produces good results. To give a chance to relatively weak extensor muscle, splinting and casting should be performed before surgery. Nonsurgical methods are not recommended for patients who have agenesis or severe hypoplasia of EPB.^14,15^ Ghani et al^10^ reported that conservative treatment was effective for patients under 1 year of age but then, and in type II and type III patients who do not respond to conservative treatment surgery gives better results. Lin et al^9^ suggested that application of a splint will give successful results in patients under 1 year of age, who have neither skin contracture at first evaluation nor absence or severe weakness of EPB. However, conservative treatment is not recommended in patients with severe hypoplasia or agenesis in whom conservative treatment is generally not successful. In cases where delaying surgery is mandatory, splinting should be performed in order to decrease severity of contractions which can cause progression of deformity. Medina et al^2^ recommended surgical treatment for patients who present after 2 years of age and who do not respond to conservative treatment.

McCarroll^11^ simply categorized this deformity into flexible and complex types. Flexible type can be treated by splinting but when skin contracture and/or ligament laxity is present surgery is necessary.

The aim of splinting and conservative management is to regain extension function of thumb. Therefore, splint should be applied for 6 months in order to keep thumb at extension and then, after active thumb extension is achieved night splints should be used for another 6 months.^10,12^ The aim of surgical treatment is to achieve near full thumb function by correcting accompanying deformities in patients who did not respond to conservative treatment. For this purpose tendon transfer in cases with absent or insufficient tendons, appropriate reconstruction in cases with tendon subluxations, several z-plasty reconstructions for first web space contraction, and thenar and adductor muscle release procedures may be performed.^10^

In conclusion, congenital clasped thumb syndrome may be confused with trigger finger. This condition should be kept in mind in cases with flexion and adduction deformity. Splinting with physical treatment is a very successful method in flexible cases or in patients who only have extensor tendon weakness. But in cases who have tendon hypoplasia or absence, who presented late, and who have contracture at first web space, it should be kept in mind that management with only splinting will generally yield unsuccessful results. As in our case, EIP tendon transfer and z-plasty of contracture in first web interval is a simple, reliable, and successful method in order to achieve functional result.
